# Small cell lung cancer progressing into fatal ascending motor and sensory polyneuropathy despite dramatic response to chemotherapy: A case report: Retraction

**DOI:** 10.1097/MD.0000000000044337

**Published:** 2025-08-22

**Authors:** Syed Mohammad Naqvi, Hashim Talib Hashim, Syed Yaseen Naqvi, Ahmed Qasim Mohammed Alhatemi, Fatimah Abdullah Sulaiman, Sally Muayad Abdulmahdi, Ahmed Dheyaa Al-Obaidi, Ashraf Fhed Mohammed Basalilah, Ammar Al-Obaidi

The article “Small cell lung cancer progressing into fatal ascending motor and sensory polyneuropathy despite dramatic response to chemotherapy: A case report”^1^, which appears in Volume 104, Issue 1 of *Medicine*, is being retracted. Information brought to our attention during a recent investigation casts doubt on both the authorship and credibility of the presented case, and the Publisher has lost faith in the integrity of the content.
